# Changes in free amino acid content and hardness of beef while dry-aging with *Mucor flavus*: Changes in the quality of beef while dry-aging with *Mucor flavus*

**DOI:** 10.1186/s40781-018-0176-6

**Published:** 2018-07-26

**Authors:** Takashi Hanagasaki, Naokazu Asato

**Affiliations:** 1 Okinawa Prefectural Industrial Technology Center, 12-2 Suzaki, Uruma City, Okinawa 904-2234 Japan; 2 Okinawa Prefectural Livestock Research Center, 2009-5 Shoshi, Nakizin Village, Okinawa 905-0426 Japan; 3 Present Address: Okinawa Prefectural Agricultural Research center, 820 Makabe, Itoman City, Okinawa 901-0336 Japan; 4 Present Address: Livestock Division of Okinawa Prefectural Government, 1-2-2 Izumizaki, Naha city, Okinawa, 900-8570 Japan

## Abstract

**Background:**

A mold strain thought to be suitable for dry-aging process was isolated. The information about the scientific aspects of molds related to dry-aging beef is scarce. We, therefore, conducted aging trials to determine the characteristics of the isolated mold strain associated with dry-aging process. Specifically, during the dry-aging of beef with the mold strain, the changes in the free amino acid content, hardness, productive loss, drip and cooking loss were analyzed. These characteristics were compared with those obtained while dry-aging in the absence of a mold.

**Results:**

The isolated mold strain was identified as *Mucor flavus*. The free amino acid content in the mold-aging beef decreased or remained constant during the aging process. However, that in the trimming sections of the beef dramatically increased in the presence of mold. In addition, hardness of mold-aging beef gradually decreased during the aging process and finally decreased significantly.

**Conclusion:**

Amino acids such as GABA (gamma-aminobutyric acid), proline, and aspartic acid were produced by our mold strain, *M. flavus* during its growth on beef meat, and the mold conferred savory odors to the dry-aged beef.

## Background

Dry-aging, an aging method, is believed to improve meat quality in terms of certain characteristics. Recently, the increased interest in dry-aged beef has emerged not only in Western countries but also in Asian countries such as South Korea, Japan, Singapore, Taiwan, and Hong Kong [4]. In Okinawa, new businesses related to the dry-aging of beef have been opened.

In Okinawa, the growth rate of gross agricultural production between 2011and 2016 was the highest in Japan. In particular, the beef industry increased by 8.5 billion yen (62.5%) over these 5 years. All of cattle born or reared in Okinawa are sold at a high price, and their meat is not easily obtained as materials for dry-aged beef. In fact, almost all dry-aged products sold by the companies in Okinawa rely on the use of imported beef. In general, imported beef can be bought at a lower price than Japanese beef. Japanese beef, particularly the type of beef called “Wagyu,” has high marbling, whereas imported beef mainly comprises lean meat with little marbling. Imported beef, therefore, has more potential in terms of improving meat quality following dry-aging.

The primary factors that determine the quality of dry-aged products include the length of aging, storage temperature, relative humidity, and air flow [4, 12]. Another important factor is the choice of intentionally incorporating certain microorganism in the process or not; if yes, then determining the type of microorganisms to be used and their culturing are also important. Owing to such factors, each company in Japan has their own method of dry-aging products.

In major cities such as New York, most of the popular dry-aged beef steak products are believed to be aged with mold. These steaks are served at high prices in top-end steakhouses and exclusive restaurants [10]. For example, “mellow and intense” and “earthy and nutty” are the types of phrases commonly used to describe the flavor characteristics of dry-aged beef [12], and these result from the type of mold used.

In general, the beef’s natural enzymes break down the proteins and connective tissue in the muscle, which leads to more tender beef [2, 3]. Moreover, reportedly, certain molds produce enzymes that enable them to penetrate into the meat, wherein they release proteases and collagenolytic enzymes that break down muscle and connective tissues [4]. There is a possibility that proteases and collagenolytic enzymes increase free amino acids. In fact, Sugioka et al. [14] reported that meat from Japanese brown cattle have high levels of some free amino acids, which increase stably during the aging process. Therefore, we hypothesized that mold-treated, dry-aged beef would exhibit increased free amino acid content and decreased meat hardness. The main mold genera associated with the dry-aging of beef are *Mucor*, *Thamnidium*, and *Rhizopus* [4].

We succeeded in isolating an excellent mold strain with robust growth on meat at low temperatures such as 2 °C. This strain released an odor similar to that of nuts and fried potato and was later identified as *M. flavus*. In this study, we analyzed the hardness and free amino acid content of beef while it was dry-aging with this mold, and we compared its characteristics with those of the meat aged in the absence of any mold.

## Methods

### Material

The meat used for the experiments were beef lumps imported from Australia. Type of Australian cow beef was crossbreed thought to be mainly in the family lineage of black cattle, which was raised in pastures. Two types of aging method were applied in each aging experiment: dry aging with mold and dry aging without mold. Six chunks of meat (approximately 6 kg of lump cut from six different animals) from the same type were grouped into the dry-aging with mold or dry-aging without mold group (further referred to as mold-aged and normal-aged, respectively), with three chunks of meat in each group. Each chunk of meat was cut and divided into five pieces for experiments involving 0, 1, 2, 3, and 4 weeks of aging.

### Mold identification

Culture and morphological observations:

A mold isolate was incubated in petri dishes with potato dextrose agar (PDA; Becton Dickinson, USA) at 15 °C for 1–3 weeks in dark condition. Microscopic slides were prepared from the portions of the colonies grown on the PDA plates by mounting them in lactophenol (with/without cotton blue). Microscopic examinations were performed with a SMZ800 stereomicroscope (Nikon, Tokyo, Japan) and a BX51 microscope (Olympus, Tokyo, Japan) with Nomarski interference contrast at magnifications of up to × 1500. All micrographs were captured with a digital camera (DS-Fi2-L3; Nikon, Tokyo, Japan).

### DNA sequencing analysis

The genomic DNA of our mold strain was extracted by physical disruption using beads (Nippon Gene Co., Ltd.). The primers used included ITS5 and ITS4 [18] for the nuclear ribosomal internal transcribed spacer (ITS) regions, which include ITS1, 5.8S, and ITS2. Polymerase chain reactions (PCRs) were performed with PrimeSTAR HS DNA polymerase (Takara Bio, Japan). The sequencing primers ITS5, ITS3, and ITS4 [18] were used for the amplification of ITS. The sequences were assembled with ChromasPro 1.7 (Technelysium Pty, Ltd., South Brisbane QLD, Australia). Multiple alignments were performed with CLUSTALW [6], and the final alignments were manually adjusted. Ambiguous positions and alignment gaps were excluded from the analysis. The neighbor-joining [11] phylogenetic tree with the Kimura two-parameter model [7] was constructed using the TechnoSuruga Lab Microbial Identification database (TechnoSuruga Laboratory, Shizuoka, Japan). A bootstrap test with 1000 iterations was used to assess the reliability of the branches [5]. The positions with gaps and the regions of uncertain nucleotide alignment were excluded from the phylogenetic analyses.

DNA extraction, PCR amplification, DNA sequencing, and molecular phylogenetic analyzes were all performed by TechnoSuruga Laboratory Co., Ltd. (Shizuoka, Japan).

### Aging environment

Aging environment was established in a refrigerator (Showa Denko K.K., Tokyo, Japan) in Okinawa Industrial Technology Center at a temperature of 2 °C. Accordingly, dry boxes were placed in the refrigerator. Further, three pieces of meat were placed in the boxes for each week under maintained conditions of approximately 80% relative humidity. For the mold-aging experiments, our mold strain was cultured on PDA plates (Merck Ltd., Tokyo, Japan) for a week and then allowed to contact each piece of meat, and these were later used for the experiments involving 1, 2, 3, or 4 weeks of aging.

### Measurement of moisture and trim losses

Moisture loss is the weight of water lost from meat and is determined by measuring the difference in meat weight between before and after it has been subjected to aging. Trim loss is weight of the trimming part of meat that is discolored and dehydrated.

### Quantitative analysis of amino acids

We first collected ≤ 1 cm-deep samples from the surface of the edible part of the beef samples at 2, 3, and 4 weeks of dry aging, after they had been trimmed. The sampling at 0 and 1 weeks was performed at a depth of > 1 cm from the surface of the meat that had not been trimmed. Sampling surface was performed on approximately 2 mm depths from the surface of the meats from both the groups on a weekly basis. These were cut and homogenized. Extract solutions were obtained from these homogenized samples after protein was removed with acetonitrile and perchlorate and fat was removed with hexane. Sample solutions for LC/MS were prepared after extract solutions had been filtered. Sample solutions were injected onto an Intrada Amino Acid column (3 × 100 mm, Imtakt Corp., Kyoto, Japan) at a flow rate of 0.6 ml/min. The separation was performed with a two-pump gradient. Solvent A was acetonitrile/tetrahydrofuran/25 mM ammonium formate/formic acid (9/75/16/0.3, *v*/v/*v*/v). Solvent B was acetonitrile/100 mM ammonium formate (20/80, v/v). The gradient program was as follows: 0, A 100%; 2.75, A 100%; 7.75, A 83%; and 7.76 min, A 0%. Analyses were monitored in the positive-ion mode using an ESI source at 350 °C and MRM.

Amino acids were sorted according to their features into four groups. Glycine, alanine, threonine, serine, and proline were classified as sweet-tasting amino acids. Aspartic acid, glutamic acid, glutamine, and asparagine were classified as umami-tasting amino acids. Methionine, lysine, isoleucine, leucine, phenylalanine, tyrosine, valine, histidine, arginine, and cystine were classified as savory-tasting amino acids. Finally, carnosine, anserine, taurine, ornithine, and GABA were classified as functional amino acids.

### Measurements of drip and cooking losses

Samples for these measurements were cut to a size of approximately 2.5 cm (length) × 2.5 cm (width) × 1 cm (height) at a depth of over 1 cm from the surface of the meat and were frozen until subsequent analyses. Prior to analyses, these frozen samples were kept at a normal temperature for 2.5 h and weighed before their drip was removed. The weight of these samples was measured again after their drip had been removed. The weight difference was calculated and was denoted as a ratio relative to the initial weight. The percentage of drip loss was calculated in this way. In terms of cooking loss, samples that had already undergone drip loss measurement were put into a plastic bag and incubated at 70 °C in a water bath for 1 h. After they had been cooled and their drip had been removed, the weight of these samples was measured. The weight difference between before and after incubation was determined as a ratio relative to the weight before incubation [9].

### Rheological properties

Breaking stress and strain of breaking point of each meat sample were measured using a rheometer RE-3305S (Yamaden Co. Ltd., Tokyo, Japan) and a breaking strength analyzer BAS-33005-16 (Yamaden Co. Ltd., Tokyo, Japan). Samples for these measurements were those that had already undergone both drip and cooking loss measurements as described above. With regard to the measurement of breaking stress, this was performed on a sample of approximately 1 cm in height using a rheometer with a plunger No. 5 stick-type at a speed of 1 mm/s. Approximately seven runs were performed for each measurement and the average was calculated.

### Statistical analysis

One-way analysis of variance (ANOVA) was used for the statistical analysis of productive loss using the JMP 13 (SAS Institute Inc., Cary, NC, USA). Tukey’s test was used to identify the differences between each week of the same aging process for each experiment. *P*-values < 0.05 were considered to be statistically significant.

## Results

### Identification of the mold strain

**Fig. 1:**
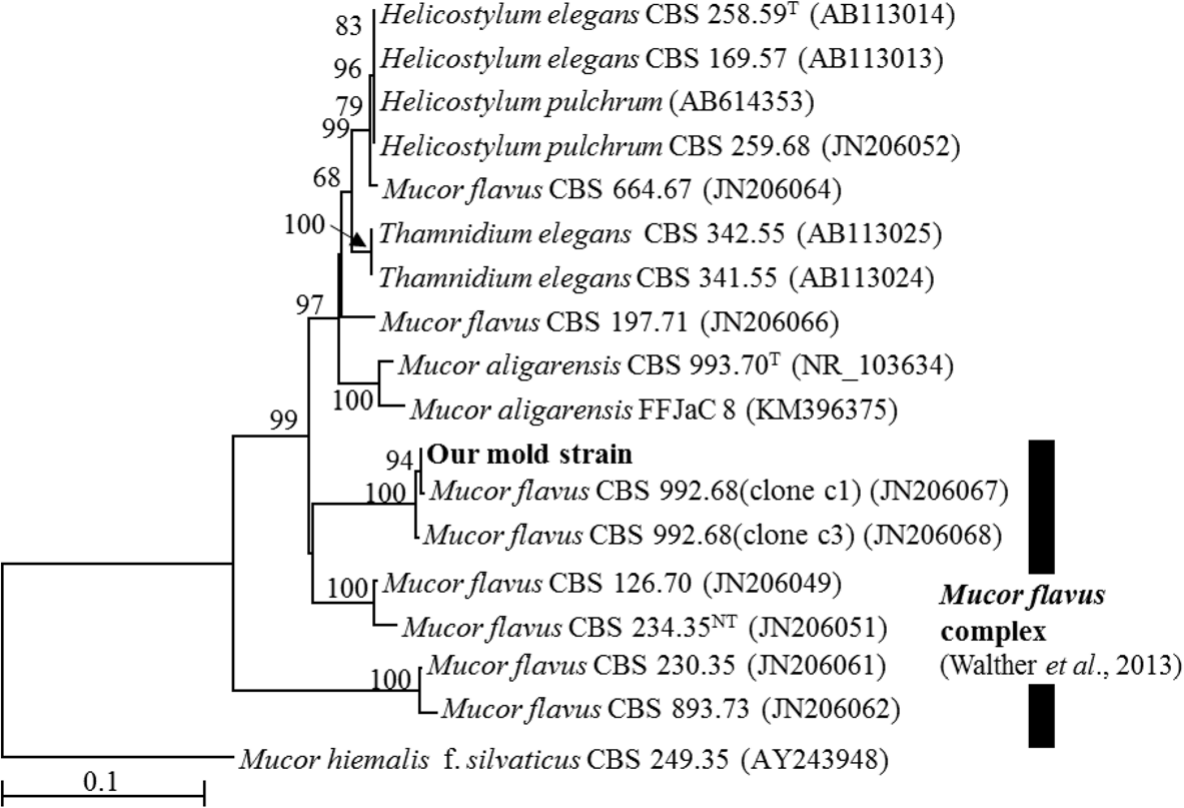
Phylogenetic relationships between our mold strain and *Mucor* species based on neighbor-joining analysis of their ITS sequences. The values on the branch nodes represent bootstrap support values (%) from 1000 iterations. Bootstrap values > 50% are indicated. The superscripts ^T^ and ^NT^ indicate ex-type and ex-neotype strains, respectively. The scale bar indicates 0.1 nucleotide substitutions/site

**Fig. 2:**
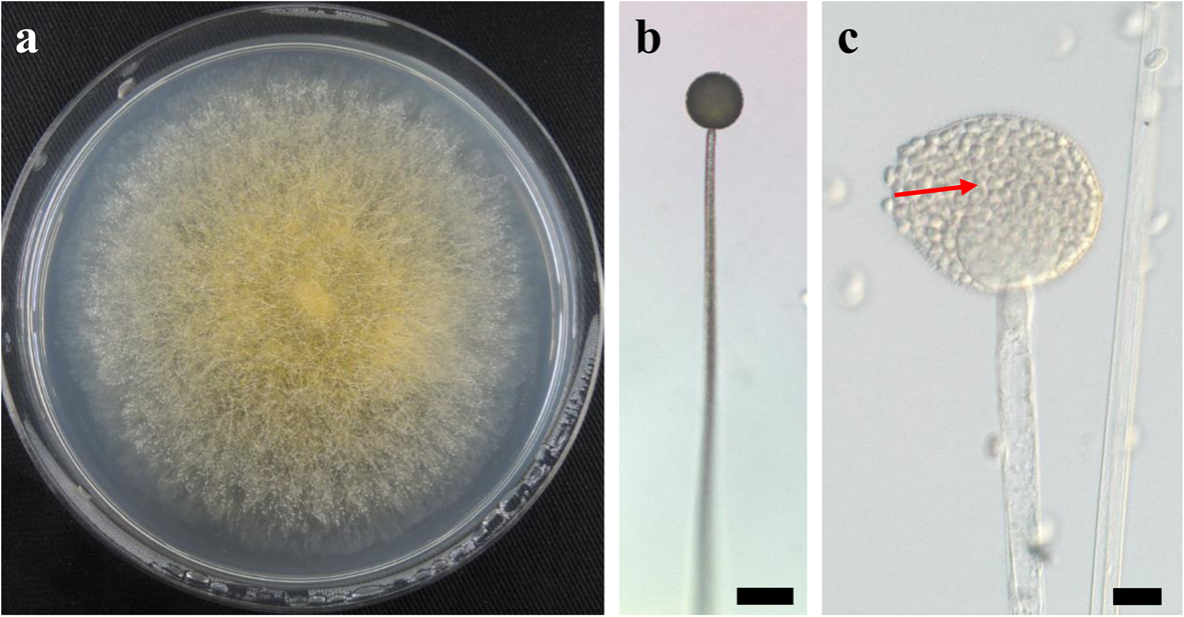
Morphological characteristics of our mold strain. **a** Colony appearance on potato dextrose agar after 1 week incubation at 15 °C. **b** and **c** Sporangiophores and sporangia with columella (red arrow). The scale bars represent 50 μm (**b**) and 10 μm (**c**)

A BLAST search showed that the ITS sequence of our mold strain had the highest sequence homology (88.2–99.5% similarity) with that of *M. flavus* in the DNA database (GenBank/DDBJ/EMBL). A neighbor-joining phylogenetic tree was constructed with the ITS sequences of our mold strain and the closely related strains according to the database. Using phylogenetic analysis, the ITS sequence of our mold strain was found to form a cluster with the *M. flavus* complex [15] (Fig. 1). Morphological observation of the mold culture colonies on PDA showed yellow to white colonies with a floccose appearance at 15 °C (Fig. 2a). Sporangiophores with multispored sporangia had erectly formed from the vegetative mycelium (Fig. 2b and c). These morphological characteristics matched those previously reported for *M. flavus* [16]. Based on its ITS sequence and morphology, our mold strain was identified as *M. flavus*.

### Changes in productive loss

**Fig. 3:**
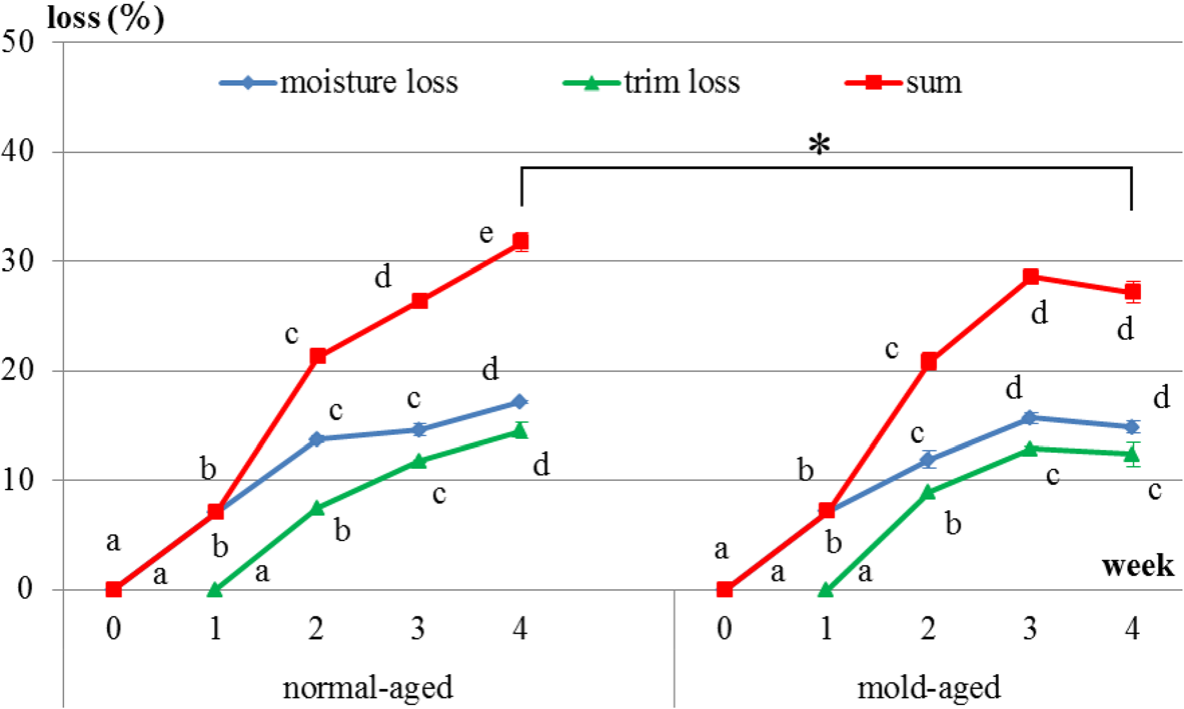
Changes in moisture and trim losses during normal- and mold-aging processes. Different letters in the same aging process represent significant differences (*p* < 0.05) between weeks. *P < 0.01 for the total productive loss between the two aging methods after 4 weeks of aging (one-way ANOVA)

Productive loss is the sum of moisture and trim losses. For both dry-aging processes (i.e., in the presence/absence of our mold strain), moisture loss occurred immediately after the initiation of dry aging, whereas trim loss occurred approximately 10 days after the initiation. In normal-aged and mold-aged beef, the moisture and trim losses increased every week (*p* < 0.001), but with no increase observed between weeks 3 and 4 for mold-aged beef (Fig. 3). Moreover, the overall productive loss after 4 weeks of aging was significantly lower for mold-aging compared with that for normal-aging (*p* < 0.01) in despite of no significant difference from 1 to 3 weeks of aging between both aging methods. The values of productive loss in normal-aged and mold-aged beef are 0, 7.0, 21.3, 26.4, 31.7 and 0, 7.2, 20.8, 28.6, 27.2 from 0, 1, 2, 3 and 4 weeks of aging.

### Changes in free amino acid content

**Fig. 4:**
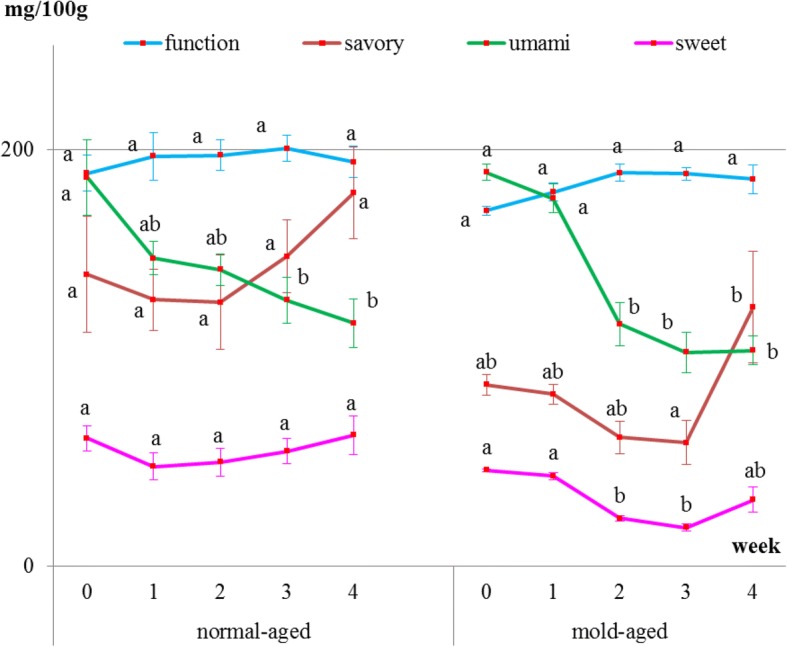
Changes in the levels of each amino acid group inside the meat during the normal- and mold-aging processes. Amino acid groups were classified as functional, sweet-, umami-, or savory-tasting. Different letters in the same aging process represent significant differences (*p* < 0.05) between weeks

Internal meat samples showed that the levels of umami-tasting amino acids decreased with both aging methods (Fig. 4 and Table 1). The level of functional amino acids did not increase but remained constant throughout both dry-aging methods. The level of sweet-tasting amino acids remained constant during the normal-aging process but decreased between weeks 2 and 3 of the mold-aging process. As for the savory-tasting amino acids, no significant changes were observed for either aging method. Additionally, aspartic acid, cystine, glycine, and GABA were not detected in any of the internal meat samples.

**Table 1:** Results of the average value ± standard deviation of each amino acid group listed in Fig. 5

Normal-aged					
Week	0	1	2	3	4
Function	189 ± 15	197 ± 20	197 ± 13	200 ± 11	194 ± 1314
Umami	186 ± 32	148 ± 14	142 ± 13	128 ± 19	116 ± 20
Sweet	5361 ± 411	5648 ± 112	5950 ± 712	6355 ± 711	10463 ± 1116
Savory	139140 ± 49	128 ± 2526	126 ± 3940	149 ± 30	179 ± 38
Mold-aged					
Week	0	1	2	3	4
Function	171 ± 4	179 ± 78	189 ± 7	188 ± 56	186 ± 12
Umami	189 ± 7	176 ± 1213	116 ± 18	102 ± 17	103 ± 12
Sweet	4746 ± 32	5043 ± 43	7523 ± 23	7218 ± 64	7832 ± 1511
Savory	87 ± 89	82 ± 8	62 ± 1314	59 ± 1819	124 ± 4647

**Fig. 5:**
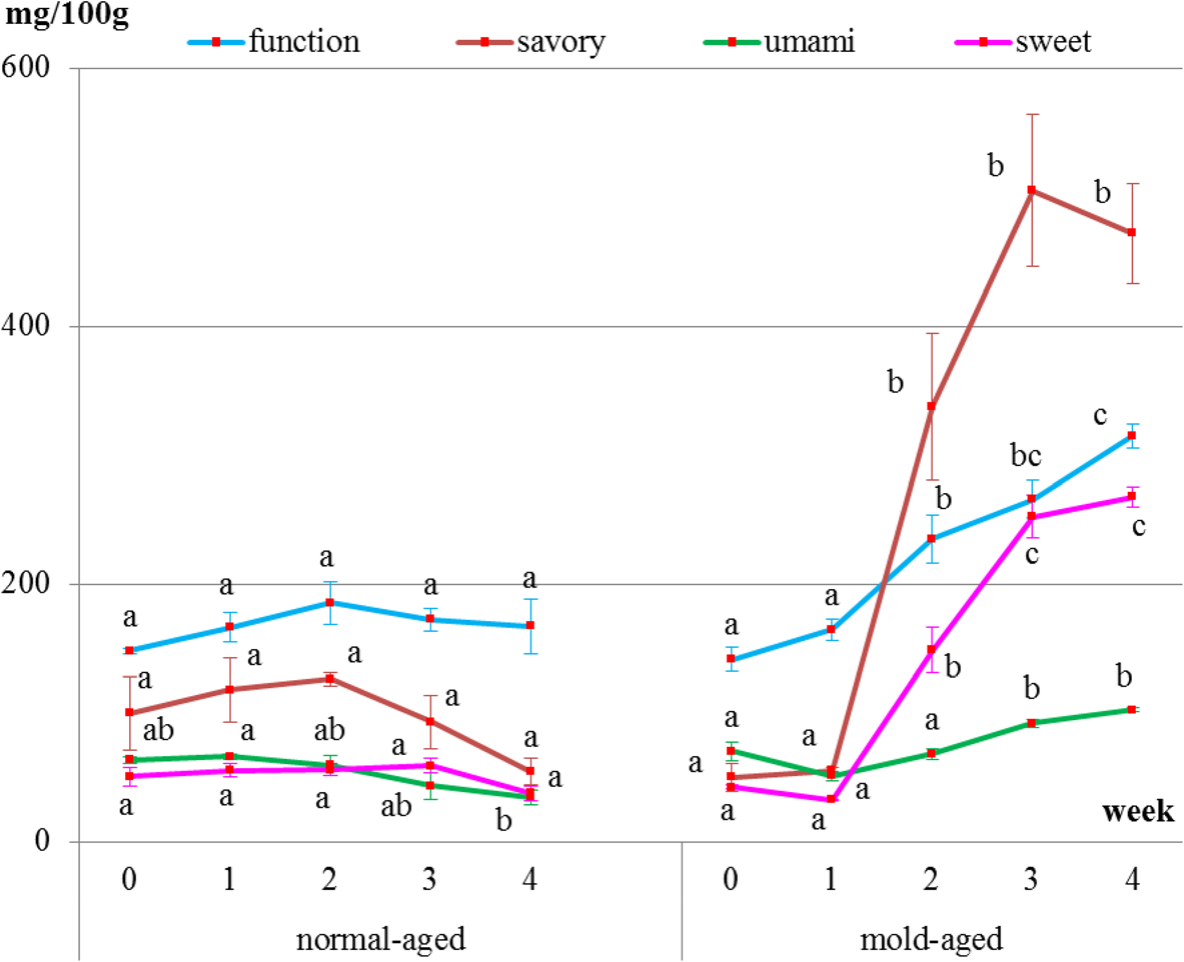
Changes in the levels of each amino acid group in surface meat during the normal- and mold-aging processes. Amino acid groups are classified as functional, sweet-, umami-, or savory-tasting. Different letters in the same aging process represent significant differences (*p* < 0.05) between weeks

**Fig. 6:**
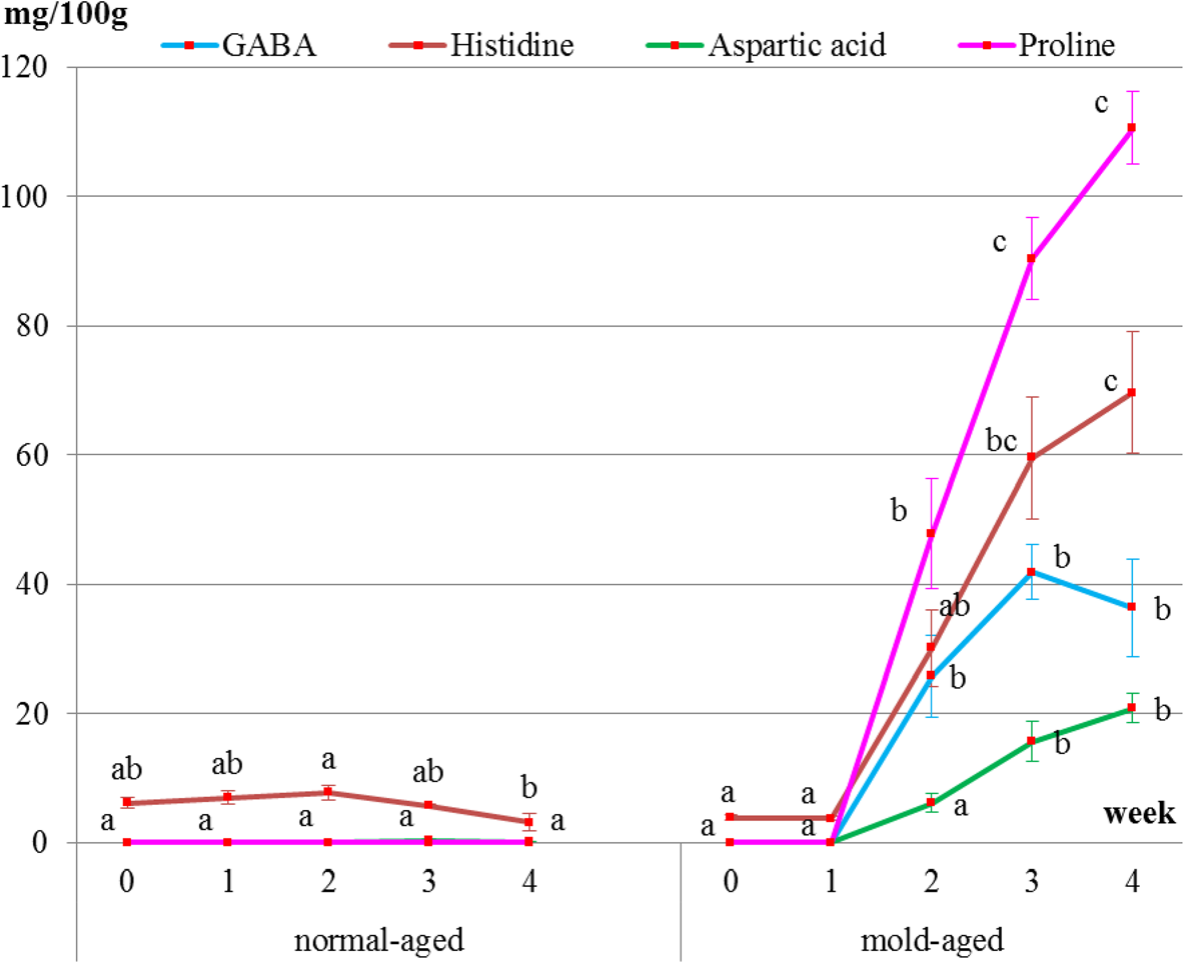
Changes in the levels of proline, aspartic acid, histidine, and GABA in surface meat during normal- and mold-aging processes. Different letters in the same aging process represent significant differences (*p* < 0.05) between weeks. GABA and aspartic acid were not detected in normal-aging beef

Surface meat sampling showed that the level of sweet-, savory-, and umami-tasting amino acids (proline, histidine, aspartic acid, and GABA, respectively) and functional amino acids dramatically increased during the mold-aging process (*p* < 0.0001), whereas it did not increase during the normal-aging process (Figs. 5 and 6, Tables 2 and 3). Proline or GABA was not detected on the surface meat of the normal-aged beef (Fig. 6 and Table 3), whereas cystine was not detected in the surface meat from either dry-aging method.

**Table 2:** Results of the average value ± standard deviation of each amino acid group listed in Fig. 6

Normal-aged					
Week	0	1	2	3	4
Function	148 ± 3 4	166 ± 20	185 ± 29 30	172 ± 16	167 ± 38
Umami	63 ± 5 6	66 ± 3 4	59 ± 13 14	43 ± 18	34 ± 10
Sweet	50 ± 12 13	55 ± 8 9	56 ± 8	59 ± 10 11	38 ± 10
Savory	100 ± 49	118 ± 42 43	126 ± 8 9	93 ± 36	54 ± 18
Mold-aged					
Week	0	1	2	3	4
Function	141 ± 16	165 ± 14 15	235 ± 32 33	266 ± 25	315 ± 15 16
Umami	70 ± 13 14	51 ± 6 7	68 ± 7	92 ± 6	103 ± 3 4
Sweet	42 ± 3	32 ± 1	149 ± 31	252 ± 29 30	268 ± 14 15
Savory	50 ± 19	55 ± 4	337 ± 98	505 ± 102 103	472 ± 68

**Table 3:** Results of the average value ± standard deviation of each amino acid listed in Fig. 7

Normal-aged					
Week	0	1	2	3	4
GABA	0 ± 0	0 ± 0	0 ± 0	0 ± 0	0 ± 0
Aspartic acid	0 ± 0	0 ± 0	0 ± 0	0 ± 1	0 ± 1
Proline	0 ± 0	0 ± 0	0 ± 0	0 ± 0	0 ± 0
Histidine	100 6± 49 2	118 7± 42 2	126 8± 8 2	93 6 ± 36 1	54 3 ± 18 3
Mold-aged					
Week	0	1	2	3	4
GABA	0 ± 0	0 ± 0	26 ± 11	42 ± 7 8	36 ± 13 14
Aspartic acid	0 ± 0	0 ± 0	6 ± 23	16 ± 56	21 ± 4
Proline	0 ± 0	0 ± 0	48 ± 15	90 ± 11	111 ± 10
Histidine	50 4 ± 19 1	55 4± 4 1	337 30 ± 98 11	505 60± 102 17	472 70± 68 17

### Changes in drip and cooking losses

**Fig. 7:**
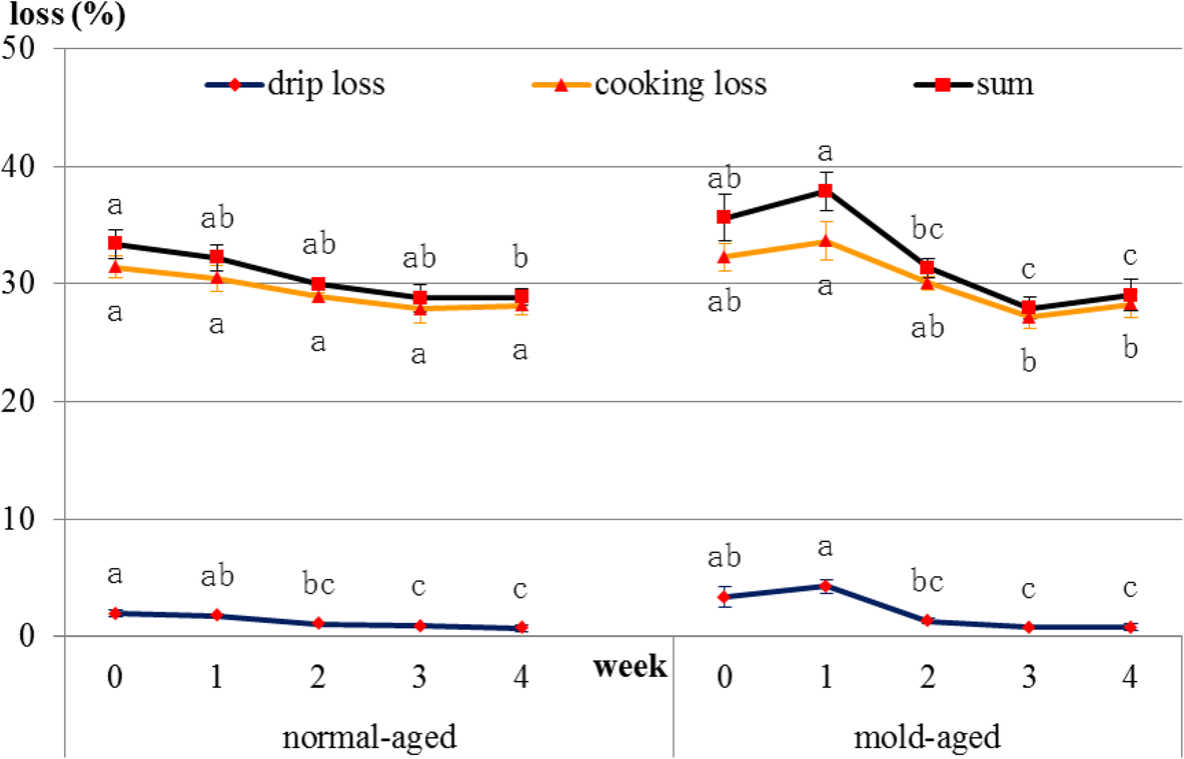
Changes in drip and cooking losses during normal- and mold-aging processes. Different letters in the same aging process represent significant differences (*p* < 0.05) between weeks

In normal-aged beef, drip loss decreased [1.9, 1.8, 1.1, 0.9, and 0.7 (weeks 0, 1, 2, 3 and 4), *p* = 0.021] and cooking loss showed a decreasing tendency [31.4, 30.5, 28.9, 27.9, and 28.2 (weeks 0, 1, 2, 3 and 4), *p* = 0.089] during the aging process. In mold-aged beef, drip loss [3.4, 4.3, 1.3, 0.7, and 0.8 (weeks 0, 1, 2, 3 and 4), *p* = 0.001] and cooking loss [32.3, 33.6, 30.1, 27.2, and 28.3 (weeks 0, 1, 2, 3 and 4), *p* = 0.013] decreased significantly during the aging process (Fig. 7).

### Changes in hardness

**Fig. 8:**
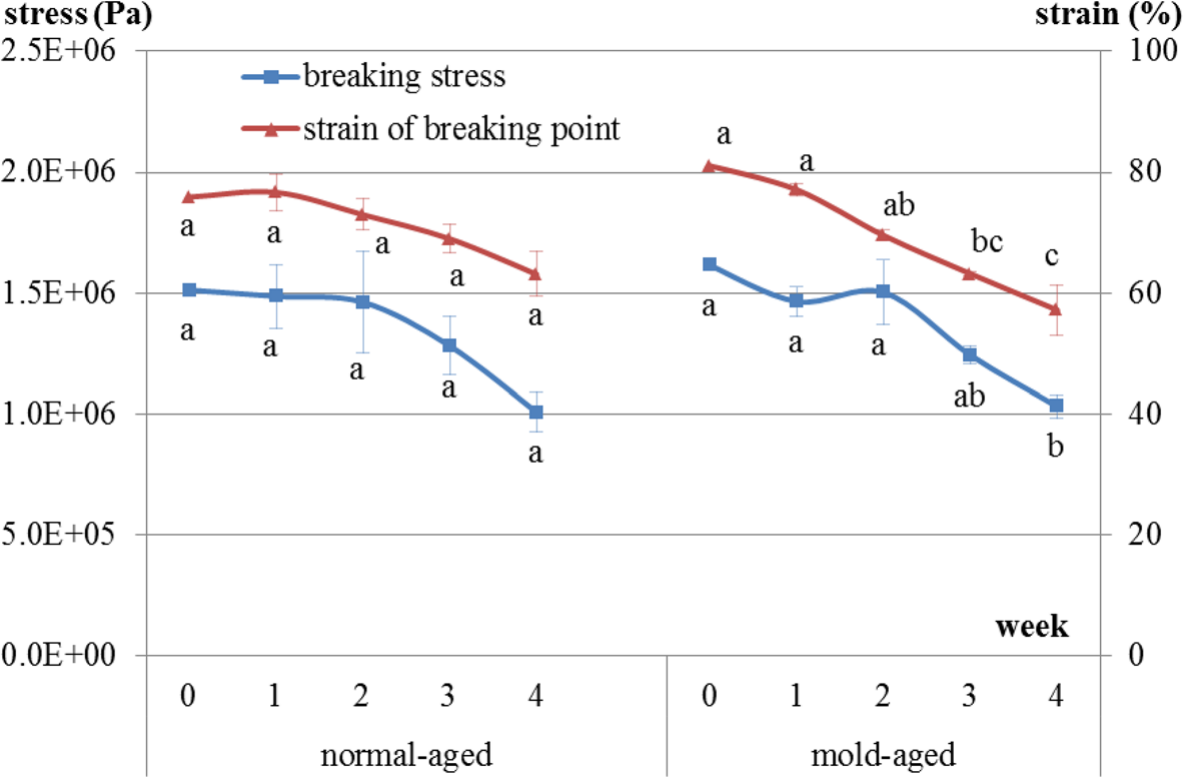
Changes in breaking stress and the strain of breaking point in beef during normal- and mold-aging processes. Different letters in the same aging process represent significant differences (*p* < 0.05) between weeks

Breaking stress in the normal-aged beef during the aging process did not significantly decrease over time [1.51E + 06, 1.49E + 06, 1.46E + 06, 1.28E + 06, and 1.01E + 06 (weeks 0, 1, 2, 3 and 4), *p* = 0.24], whereas for mold-aged beef, it did significantly decrease [1.61E + 06, 1.47E + 06, 1.50E + 06, 1.24E + 06, and 1.03E + 06 (weeks 0, 1, 2, 3 and 4), *p* = 0.008] (Fig. 8). The strain of the breaking point had the same decreasing tendency in both groups but was only significant for the mold-aged beef [normal-aging: 75.9, 76.7, 73.0, 69.0, and 63.1 (weeks 0, 1, 2, 3 and 4), *p* = 0.1693; mold-aging: 81.1, 77.1, 69.6, 63.2, and 57.2 (weeks 0, 1, 2, 3 and 4), *p* = 0.0003]. The breaking stress values were similar at the endpoints of the two aging methods.

## Discussion

Our mold strain was found to belong to the *Mucor* genus, which is used for preparing various food products, including sausage, salami, cheese, tofu, and vegetables, worldwide [13]. Therefore, *Mucor* is a relatively safe mold genus, and its fermentation effects are expected to improve the quality of meat in terms of certain aspects.

**Fig. 9:**
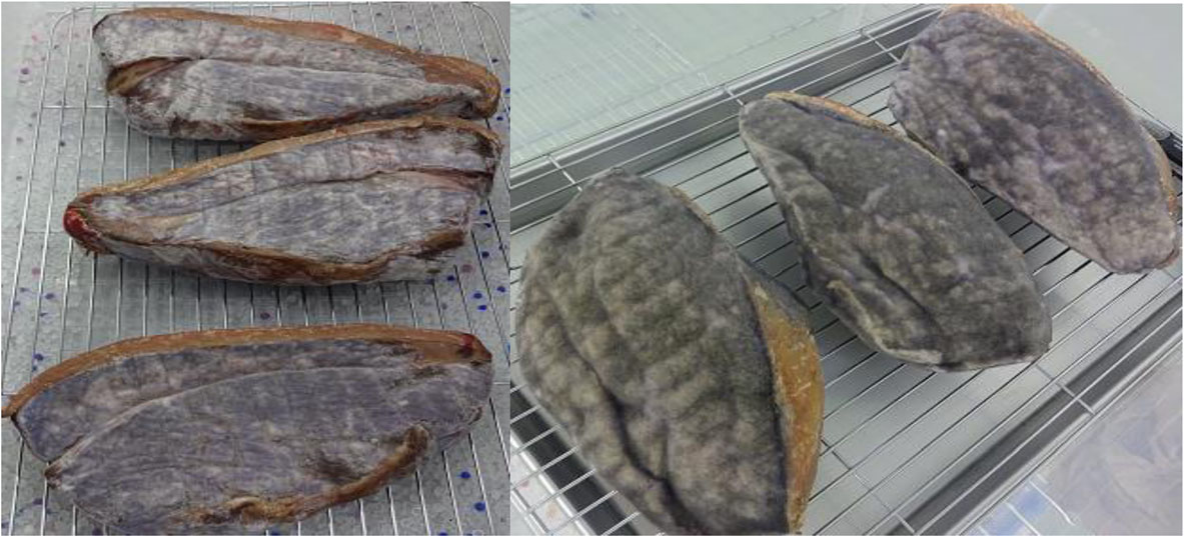
Changing appearance of mold-aged beef. The photographs of mold-aged beef following two (left) and three (right) weeks of aging

In the present study, during mold-aging process, the mold mycelium was observed to sparsely spread on the meat during the first week and to completely cover it after 2 weeks, producing light gray-colored meat and the mold grew stably even after that (Fig. 9). While it grew, productive loss did not increase between the weeks 3 and 4 of aging and did not exceed 30%, a significantly lower level than that in normal-aged beef. This was an interesting finding, and the mold strain possibly prevented moisture evaporation from the meat and trim once it had completely covered the meat surface. Substantial yield losses and increased processing times that are associated with the dry aging process require to increase prices for the sale and distribution of dry-aged product [8]. Decreasing productive loss of dry-aged beef is very important because it directly influence the retail segment of the beef industry. Under the circumstances, there is a possibility that covering the meat by the mold increases retail yield and it may be the key of dry aging process.

The amino acid content of the interior meat samples did not stably increase during either aging process. Instead, umami-tasting amino acid levels were observed to decrease because glutamine which accounts for a large percentage of umami-tasting amino acid, levels decreased sharply during the aging process. Savell reported that many of the compounds responsible for flavor are concentrated by the dry-aging process [12]. Namely, the distinguishing effect of the dry-aging process on beef is that it concentrates the flavor [4, 12, 17]. Contrary to expectation, the present result didn’t show that free amino acids concentrate during the aging process as reported above. It, however, is difficult to conclude the changes in each amino acid based on only the dry-aging process considering the different lengths of time between slaughter and the initiation of dry-aging for the different pieces of meat; this would greatly influence the concentration of each amino acid present.

It is particularly interesting that the amino acids increased on the meat surface during the mold-aging process but not during the normal-aging process. GABA, one of the functional amino acids, that is famous for its sedative and other effects [1] significantly increased after 2 weeks of mold-aging, when the mold completely covered the surface meat. To the best of our knowledge, there have been no previous reports of GABA content in beef meat. It is, therefore, highly possible that our mold strain itself produced GABA, proline, and aspartic acid during growth.

The hardness of mold-aging beef gradually decreased during the aging process and had significantly decreased after 4 weeks of aging. Proteases and collagenolytic enzymes produced by certain molds have previously been shown to break down the muscle and connective tissues of meat [4]. In contrast, the hardness of normal-aging beef did not significantly decrease. However, a definitive conclusion that only mold-aged beef became more tender cannot be drawn from the study findings. No significant decrease was observed during the normal-aging process due to the large error margins in the data. It is possible that normal-aged beef becomes softer as the natural enzymes in beef have been reported to break down proteins and connective tissue in the muscle, leading to more tender beef [2, 3]. Notably, the breaking stress values were similar at the endpoints of the two aging methods in our study.

The present study showed that our isolated mold strain mainly had an effect on the free amino acid content and beef hardness. During the aging process, the mold was observed to also produce savory odors, which are important factors in increasing meat flavor and its appeal. Therefore, further studies are needed to determine the chemical compounds conferring this odor to mold-aged beef.

## Conclusion

Amino acids such as GABA, proline, and aspartic acid were produced by our isolated mold strain, *M. flavus* during its growth on beef meat, and the mold conferred savory odors to the dry-aged beef.
