# Correction to: Changes in free amino acid content and hardness of beef while dry-aging with *Mucor flavus*

**DOI:** 10.1186/s40781-018-0179-3

**Published:** 2018-09-14

**Authors:** Takashi Hanagasaki, Naokazu Asato

**Affiliations:** 1 Okinawa Prefectural Industrial Technology Center, 12-2 Suzaki, Uruma City, Okinawa 904-2234 Japan; 2 Okinawa Prefectural Livestock Research Center, 2009-5 Shoshi, Nakizin Village, Okinawa 905-0426 Japan; 3 Present Address: Okinawa Prefectural Agricultural Research center, 820 Makabe, Itoman City, Okinawa 901-0336 Japan; 4 Present Address: Livestock Division of Okinawa Prefectural Government, 1-2-2 Izumizaki, Naha city, Okinawa 900-8570 Japan

## Correction

Following publication of the original article [1], the authors reported that the modifications they requested to the data in Tables 1, 2 and 3 were incorrectly implemented due to a misunderstanding in mark-ups. Also, the titles of the tables should be modified as follows:

**Table 1:** Results of the average value ± standard deviation of each amino acid group listed in Fig. 4

Normal-aged					
Week	0	1	2	3	4
Function	189 ± 15	197 ± 20	197 ± 13	200 ± 11	194 ± 14
Umami	186 ± 32	148 ± 14	142 ± 13	128 ± 19	116 ± 20
Sweet	61 ± 11	48 ± 12	50 ± 12	55 ± 11	63 ± 16
Savory	140 ± 49	128 ± 26	126 ± 40	149 ± 30	179 ± 38
Mold-aged					
Week	0	1	2	3	4
Function	171 ± 4	179 ± 8	189 ± 7	188 ± 6	186 ± 12
Umami	189 ± 7	176 ± 13	116 ± 18	102 ± 17	103 ± 12
Sweet	46 ± 2	43 ± 3	23 ± 3	18 ± 4	32 ± 11
Savory	87 ± 9	82 ± 8	62 ± 14	59 ± 19	124 ± 47

**Table 2:** Results of the average value ± standard deviation of each amino acid group listed in Fig. 5

Normal-aged					
Week	0	1	2	3	4
Function	148 ± 4	166 ± 20	185 ± 30	172 ± 16	167 ± 38
Umami	63 ± 6	66 ± 4	59 ± 14	43 ± 18	34 ± 10
Sweet	50 ± 13	55 ± 9	56 ± 8	59 ± 11	38 ± 10
Savory	100 ± 49	118 ± 43	126 ± 9	93 ± 36	54 ± 18
Mold-aged					
Week	0	1	2	3	4
Function	141 ± 16	165 ± 15	235 ± 33	266 ± 25	315 ± 16
Umami	70 ± 14	51 ± 7	68 ± 7	92 ± 6	103 ± 4
Sweet	42 ± 3	32 ± 1	149 ± 31	252 ± 30	268 ± 15
Savory	50 ± 19	55 ± 4	337 ± 98	505 ± 103	472 ± 68

**Table 3:** Results of the average value ± standard deviation of each amino acid listed in Fig. 6

Normal-aged					
Week	0	1	2	3	4
GABA	0 ± 0	0 ± 0	0 ± 0	0 ± 0	0 ± 0
Aspartic acid	0 ± 0	0 ± 0	0 ± 0	0 ± 1	0 ± 1
Proline	0 ± 0	0 ± 0	0 ± 0	0 ± 0	0 ± 0
Histidine	6 ± 2	7 ± 2	8 ± 2	6 ± 1	3 ± 3
Mold-aged					
Week	0	1	2	3	4
GABA	0 ± 0	0 ± 0	26 ± 11	42 ± 8	36 ± 14
Aspartic acid	0 ± 0	0 ± 0	6 ± 3	16 ± 6	21 ± 4
Proline	0 ± 0	0 ± 0	48 ± 15	90 ± 11	111 ± 10
Histidine	4 ± 1	4 ± 1	30 ± 11	60± 17	70± 17

- Table 1 Results of the average value ± standard deviation of each amino acid group listed in Fig. 5 → 4
- Table 2 Results of the average value ± standard deviation of each amino acid group listed in Fig. 6 → 5
- Table 3 Results of the average value ± standard deviation of each amino acid listed in Fig. 7 → 6

The original article has been corrected.

The correct versions of the tables have been included in this correction.
